# *In silico* investigation of the genus *Campylobacter* type VI secretion system reveals genetic diversity in organization and putative effectors

**DOI:** 10.1099/mgen.0.000898

**Published:** 2022-10-31

**Authors:** Luca Robinson, Janie Liaw, Zahra Omole, Nicolae Corcionivoschi, Abderrahman Hachani, Ozan Gundogdu

**Affiliations:** ^1^​ National Heart and Lung Institute, Imperial College London, London, UK; ^2^​ Faculty of Infectious and Tropical Diseases, London School of Hygiene & Tropical Medicine, London, UK; ^3^​ Bacteriology Branch, Veterinary Sciences Division, Agri-Food and Biosciences Institute, Belfast, UK; ^4^​ Bioengineering of Animal Resources, University of Life Sciences – King Mihai I of Romania from Timisoara, Timisoara, Romania; ^5^​ The Peter Doherty Institute for Infection and Immunity, Department of Microbiology and Immunology, University of Melbourne, Melbourne, VIC, Australia

**Keywords:** *Campylobacter*, effectors, pathogenicity island, T6SS organization, type VI secretion system (T6SS)

## Abstract

Bacterial type VI secretion systems (T6SSs) are contractile nanomachines that deliver proteinic substrates into target prokaryotic or eukaryotic cells and the surrounding milieu. The genus *Campylobacter* encompasses 39 recognized species and 13 subspecies, with many belonging to a group known as ‘emerging *Campylobacter* pathogens’. Within *Campylobacter *, seven species have been identified to harbour a complete T6SS cluster but have yet to be comparatively assessed. In this study, using systematic bioinformatics approaches and the T6SS-positive *Campylobacter jejuni* 488 strain as a reference, we explored the genus-wide prevalence, similarity and make-up of the T6SS amongst 372 publicly available ‘complete’ *Campylobacter* genomes. Our analyses predict that approximately one-third of *Campylobacter* species possess a T6SS. We also putatively report the first identification of a T6SS in four species: *Campylobacter cuniculorum, Campylobacter helveticus, Campylobacter armoricus* and *Campylobacter ornithocola *. The *Campylobacter* T6SSs cluster into three distinct organizations (I–III), of which two break down into further variants. Thirty T6SS-containing genomes were found to harbour more than one *vgrG* gene, with *Campylobacter lari* strain NCTC 11845 possessing five. Analysis of the *C. jejuni* Pathogenicity Island-1 confirmed its conservation amongst T6SS-positive *C. jejuni* strains, as well as highlighting its diverse genetic composition, including additional putative effector–immunity pairs (e.g. PoNe and DUF1911 domains). Effector–immunity pairs were also observed neighbouring *vgrG*s in several other *Campylobacter* species, in addition to putative genes encoding nucleases, lysozymes, ATPases and a ferric ATP-binding cassette uptake system. These observations highlight the diverse genetic make-up of the T6SS within *Campylobacter* and provide further evidence of its role in pathogenesis.

## Data Summary

All genomes used in this study were retrieved from the National Center for Biotechnology Information RefSeq genome database (www.ncbi.nlm.nih.gov/refseq) and metadata collected from the NCBI BioSample database (www.ncbi.nlm.nih.gov/biosample) (accession numbers and metadata available in Table S1, available in the online version of this article), except for *C. jejuni* strain 488 for which the raw fastq files were previously deposited in ENA under the study accession PRJEB41135, and the assembled gbk file can be found in our previous publication [1].

**Impact Statement**

The T6SS is an important arbitrator of bacterial survival. Its contribution to virulence and survival in the foodborne gastroenteritis pathogen *Campylobacter jejuni* is now being revised. Therefore, there is an incentive to determine its prevalence and composition amongst the genus *Campylobacter *, especially in those of the ‘emerging *Campylobacter* pathogen’ group which opportunistically infect both humans and animals. Horizontal gene transfer is a common means bacterial species use to spread genes, promoting survival in competitive and non-ambient environments. The extent to which this has contributed to the translocation of T6SS genes amongst *Campylobacter* species has been poorly described thus far. By analysing the genomes of publicly available *Campylobacter* species, we describe a belated analysis of the presence and genetic make-up of the T6SS within the genus. We report intra- and interspecific similarities and differences of the T6SS, as well as putative substrates that could be secreted by the apparatus. By conveying T6SS-specific spread, especially within characterized pathogenic species, future studies will be able to investigate its contribution to virulence and identify therapeutic targets to combat infection.

## Introduction

Bacteria inhabit a multitude of environments where conflict engagement and resistance to stresses drive adaptation. To subsist in these hostile conditions, bacteria have established multiple physiological mechanisms to promote fitness. Bacterial secretion systems are a ubiquitous apparatus of protein transportation essential for interaction with the surroundings and neighbouring cells [2]. In gram-negative bacteria, they are categorized into Types I–X [2–4]. The type VI secretion system (T6SS) is a transmembrane proteinaceous nanomachine found in ~25 % of *Proteobacteria* and contributes roles to interbacterial enmity, host–pathogen interaction, resistance to environmental stresses, natural transformation and endurance in competitive ecosystems [5–7]. Secretion of a panel of T6SS-specific effectors into target cells and the presence of cognate immunity proteins to prevent autotoxicity mediate these hostile interactions, ensuring kin protection and hindrance of competitors [5, 8]. Hence, effectors have been characterized to possess both antibacterial [9–11] and anti-eukaryotic activity [12].

Consisting of approximately 14 core components [13], the T6SS is abundant within gram-negative bacteria [14, 15]. Homologous features to the bacteriophage T4 tail-, baseplate- and membrane-like spanning structures assemble to form a bacterial contractile apparatus [16, 17]. Proteins TssJ, TssL and TssM interact to form a complex which anchors the secretion system to the membrane. The cytoplasmic baseplate (TssEFGK) of the apparatus then binds to the membrane complex, as does the contractile sheath (composed of TssB and TssC). A needle-like tube consisting of hexameric rings of the protein Hcp/TssD is then encased by the sheath and capped by the protein TssA at the opposing cellular membrane [13, 18]. The machinery also possesses a puncturing spike composed of trimers of the protein VgrG, a homologue of the phage spike proteins gp27/gp5 [17, 19]. This spike is further sharpened by a proline–alanine–alanine–arginine (PAAR)-repeat protein [20]. Upon contraction, the Hcp tube, VgrG-PAAR complex and any associated effectors are propelled across the bacterial membrane into adjacent target cells (with penetration facilitated by the spike) or the external environment [5]. Disassembly of the system is coordinated by the ATPase ClpV, a component that has not been identified to date in T6SS-harbouring *Campylobacter* species [18, 21–23]. Accessory proteins, such as the post-translational regulator Fha1 (TagH in *Campylobacter jejuni *), also contribute important roles to T6SS assembly and secretion of effectors [24–26]. Recognition of associated proteins can occur through the presence of motifs [20, 27], yet due to the lack of a widely distinguishable feature amongst secreted substrates, effectors are generally categorized into two classes: ‘cargo’ or ‘specialized’ [5]. The former represents effectors which covalently interact with one or two of the components of the cellular device before secretion [24, 28], whilst the latter exist as toxin domain-containing extensions of secreted structural components [25, 26].

The genus *Campylobacter* encompasses a collection of 44 proposed species, of which 39 names have been published and recognized by the International Code of Nomenclature of Prokaryotes, and 13 subspecies observed to colonize a range of environments [29–32]. This ability to ubiquitously inhabit diverse environmental conditions can frequently lead to infection in humans, known as campylobacteriosis [30]. *Campylobacter* is the leading cause of bacterial foodborne gastroenteritis worldwide, with *C. jejuni* as the causative agent of the majority of infections in humans and is therefore of significant clinical relevance [30, 33]. Several *Campylobacter* species belong to a group known as ‘emerging *Campylobacter* pathogens’, including *Campylobacter concisus, Campylobacter fetus* and *Campylobacter lari,* which opportunistically infect both animals and humans [29, 34, 35]. A small proportion of these documented *Campylobacter* species still possess only one available genomic sequence, such as *Campylobacter ornithocola* and *Campylobacter rectus* [29]. In comparison, *C. jejuni* has over 200 genomes of quality level ‘complete’ in the National Center for Biotechnology Information (NCBI) RefSeq database [36]. Within *Campylobacter *, several species are described to harbour a T6SS, with the earliest reported and predominantly investigated in *C. jejuni* [25, 36–40]. Studies have revealed the association of the *C. jejuni* T6SS to numerous physiological functions, including pathogenic interaction (colonization, invasion and adhesion), cytotoxicity and resistance to oxidative stress [25, 41]. Recently, we conducted a large comparative analysis of the T6SS in a population of publicly available *C. jejuni* genomes, determining the presence of a *Campylobacter jejuni* Integrated Element 3 (CJIE3)-variant termed *Campylobacter jejuni* Pathogenicity Island-1 (CJPI-1) harbouring the T6SS amongst a significant proportion of strains [1]. Other studies have also conducted similar taxonomic-scale analyses [38, 42], as well as briefly comparing the T6SS operon organization between species [25, 43]. Further, T6SS-harbouring genomic islands, integrative elements and plasmids have also been identified within several *Campylobacter* species [40, 44].

While several studies have called attention to the existence and importance of the T6SS in a growing number of pathogenic *Campylobacter* species, its (i) evolutionary origins, (ii) genus-wide prevalence, (iii) interspecific similarity, (iv) intraspecific genetic transfer, (v) range of associated effectors and (vi) contribution to pathogenesis remain largely unknown. We have therefore performed a bioinformatic analysis of 372 publicly available *Campylobacter* genomes encompassing 33 species and 17 unassigned strains, investigating the prevalence, genetic composition and make-up of the T6SS. The major T6SS components TssC, Hcp and VgrG, and a T6SS-harbouring genetic element were investigated using the T6SS-positive *C. jejuni* 488 strain as a reference. A systematic prediction of genes neighbouring identified *vgrG*s was also conducted. We identified several potentially insightful inter- and intraspecific similarities and differences between T6SS-harbouring genomes, thus expanding our current understanding of the T6SS diversity and roles.

## Methods

### *In silico* identification of T6SS-containing *Campylobacter* genomes

Nucleotide and protein sequences of *Campylobacter* genomes were collected from the NCBI RefSeq genome database release 205 (March 2021) at assembly level ‘complete genome’ or higher [36]. The T6SS-positive *C. jejuni* 488 strain (a human isolate from Brazil) [1] was included in the genome dataset as a reference, and a local nucleotide and protein database was constructed from a total of 372 genomes. Metadata, including host and sample location, was collected from the NCBI BioSample database [45].

blastp (blast+ v2.12.0) [46, 47] was employed to identify the 13 T6SS components amongst the *Campylobacter* genomes, using an expected value (E-value) of 1e-10. Amino acid sequences of the 14 T6SS loci (two VgrGs) from the T6SS-positive *C. jejuni* 488 strain [1] were aligned against a local protein dataset created for the *Campylobacter* genomes. A similarity percentage was calculated by dividing the bit-score value for each amino acid alignment by twice the specific lengths of the individual query amino acid sequence to facilitate T6SS loci identification similarly to that reported previously [48]. Genome visualization was performed in Artemis (v14.0.12) to manually inspect the T6SS operon organization [49] and comparative visualization was conducted using Clinker (v0.0.23) [50], with default parameters. Domain prediction was conducted using NCBI CDD-blast [51], with default parameters.

### Phylogenetic analysis of all *Campylobacter* genomes under study

To construct a phylogenetic tree of all *Campylobacter* genomes in the local dataset, blastp [46] was employed to identify and extract the protein AtpA from all genomes. AtpA (ATP synthase F1 complex alpha subunit) is used to sequence type *C. jejuni* and *Campylobacter coli* isolates by PubMLST [52] and is therefore commonly used to reconstruct phylogenetic trees of the genus *Campylobacter* [53, 54]. The protein sequence of CJ488_00072 (AtpA homologue) was first aligned against the local *Campylobacter* protein database. Then, extracted sequences were aligned using muscle [55], with default parameters. A maximum-likelihood tree was then reconstructed with FastTree2 (v2.1.10) [56] using the alignment file and WAG+CAT model parameters. The tree was visualized using the Interactive Tree of Life (iTOL, v6) webtool (https://itol.embl.de) [57].

### Phylogenetic analysis of TssC and Hcp components

blastp [46] was employed to identify and extract the components TssC and Hcp amongst the local *Campylobacter* genomes, using an E-value=1e-10. Protein sequences CJ488_00968 (Hcp) and CJ488_00974 (TssC) from *C. jejuni* 488 were aligned against the local *Campylobacter* protein database. Extracted sequences were then aligned using muscle [55] with default parameters. A phylogenetic tree was reconstructed from the alignment file for each component using the maximum-likelihood method, with JTT modelling, partial deletion (95 %) and bootstrapping (*n*=500) parameters, conducted in the Molecular Evolutionary Genetics Analysis X (megax, v10.2.6) software package [58]. These analyses contained 62 amino acid sequences, respectively. Tree display was conducted using the iTOL (v6) webtool [57].

### Prediction of VgrG proteins harboured in *Campylobacter* genomes

blastp [46] was employed to identify homologous VgrG proteins amongst the *Campylobacter* genomes, using an E-value=1e-10. The N-terminal amino acid sequence of VgrG1 and VgrG2 possesses >99 % amino acid identity [1], and therefore only the N-terminal amino acid sequence of VgrG1, containing the VgrG domain (COG3501), from *C. jejuni* 488 was aligned against the local *Campylobacter* protein database to identify VgrG homologues. Sequences were then filtered to remove any of the traits: (i) sequence matched to a genome that was not considered to harbour a T6SS (e.g. *C. jejuni* CLB104), (ii) possessed a fragmented VgrG ORF (e.g. AEI23_RS09810 and AEI23_RS05295 from *C. jejuni* CJ018CCUA), (iii) internal stop codons present in the sequence (e.g. EL232_RS07845 from *C. jejuni* NCTC 13265), (iv) missing methionine start to the sequence, (v) less than 100 aa in length, and/or (vi) the VgrG domain was not identified using NCBI-CDD blast (e.g. AEI20_RS09140 from *C. jejuni* CJ071CC464). A total of 19 sequences were removed from further analysis. Domain prediction was conducted using NCBI-CDD [51] with default parameters.

### *In silico* identification of CJPI-1-containing genomes

To identify the presence of CJPI-1 amongst the *C. jejuni* genomes from the local *Campylobacter* dataset, the CJPI-1 genes *CJ488_0930* (CJPI-1 integrase), *CJ488_0941* and *CJ488_1004* from *C. jejuni* 488 were used in an *in silico* identification method as proxies [1]. *CJ488_0941* and *CJ488_1004* are homologues of *C. jejuni* RM1221 strain genes *cje1105* and *cje1153*, respectively, harboured in the CJIE3. blastn [46] was employed, using default parameters, to align the nucleotide sequence of *CJ488_0930*, *CJ488_0941* and *CJ488_1004* against the local nucleotide dataset created for the *Campylobacter* genomes. A similarity percentage was calculated according to Fridman *et al.* [48]. To be regarded as positive for CJPI-1, a minimum similarity of 50 % was required for two of the three genes: *CJ488_0930*, *CJ488_0941* and/or *CJ488_1004*. To be regarded as possessing a T6SS-harbouring plasmid, (1) a minimum similarity of 50 % was required to just the gene *CJ488_0930* and (2) the presence of at least 12 T6SS loci (T6SS-positive). Comparative genomic analysis of CJPI-1-harbouring *C. jejuni* genomes was conducted using the Artemis Comparison Tool (v18.1.0) [59]. Query cover was calculated using the blastn (megablast) webtool [46], with the nucleotide sequence of CJPI-1 from *C. jejuni* 488 acting as the query and the predicted CJPI-1 sequence in the other *C. jejuni* strain as the subject.

### Functional prediction of genes downstream and upstream of *vgrG*s harboured by T6SS-positive *Campylobacter* genomes

Genes neighbouring *vgrG*s are commonly identified to possess toxic activity and are characterized as cognate effectors [24, 60]. Protein sequences for genes identified as one to five genes upstream (+5) or one to five genes downstream (−5) of *vgrG* genes identified in the previous analysis stage (94 sequences) were systematically extracted and collated. Amino acid sequences were then submitted to the NCBI CDD-blast (default parameters, concise results) [51], SignalP (v5.0, parameter – Gram-negative) [61] and TMHMM (v2.0, default parameters) [62] servers for functional prediction.

### *In silico* identification of PAAR-like domain-containing sequences

blastp [46] was employed to identify PAAR-like domain-containing proteins [1] amongst the *Campylobacter* genomes, using an E-value=1e-10. The amino acid sequence of the PAAR-like domain (amino acids 25–150) from CJ488_00990 in *C. jejuni* 488 was aligned against the local *Campylobacter* protein database. Homologous sequences were then subject to domain prediction using NCBI CDD-blast and Pfam [51, 63], with default parameters.

Identified PAAR-like domain-containing protein sequences were then aligned using ClustalOmega [64] and visualized with WebLogo3 [65], with default parameters, to confirm the presence of conserved cysteine and histidine residues.

## Results

### One-third of *Campylobacter* species in our local database harbour a T6SS

**Fig. 1.**
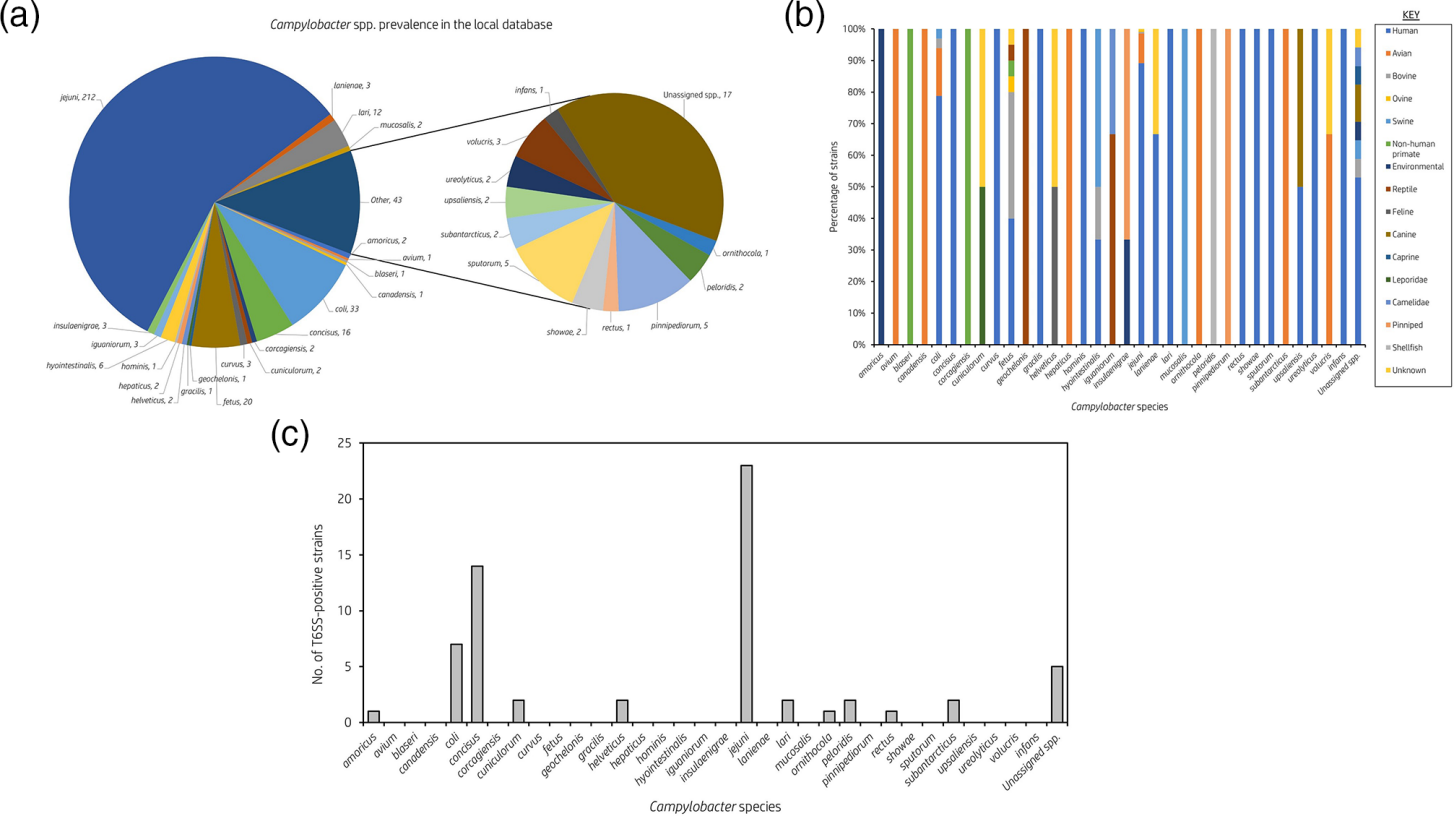
Dataset characteristics for the analysed *Campylobacter* species under study. (**a**) Pie chart showing the distribution of *Campylobacter* species within our local database. The number of isolates per species in our dataset is given next to the species name. (**b**) Bar graph showing the percentage distribution of isolation source for each *Campylobacter* species within our local database. (**c**) Bar graph showing the prevalence of T6SS-positive strains within the *Campylobacter* species in our dataset.

To estimate the prevalence of the T6SS amongst the genus *Campylobacter *, we first constructed a local database of *Campylobacter* genomes of assembly level ‘complete genome’ or higher (*n*=371) from the NCBI RefSeq database [36]. This was further populated with the T6SS-positive *C. jejuni* 488 strain as a reference, creating a total of 372 genomes (Table S1). Our local database consisted of 33 *Campylobacter* taxa and 17 strains unassigned to any known species. Genome size ranged from 1.465 Mbp (* Campylobacter insulaenigrae* NCTC 12 927) to 2.572 Mbp (* C. rectus* ATCC 33 238). *C. jejuni* was the most abundant species in our database*,* contributing 212 genomes to the dataset, followed by *C. coli* (*n*=33) and *C. fetus* (*n*=20) (Fig. 1a). Metadata was also collected, of which the two most prevalent sources of isolation were humans (*n*=281) followed by avian species (*n*=34) (Fig. 1b).

**Fig. 2.**
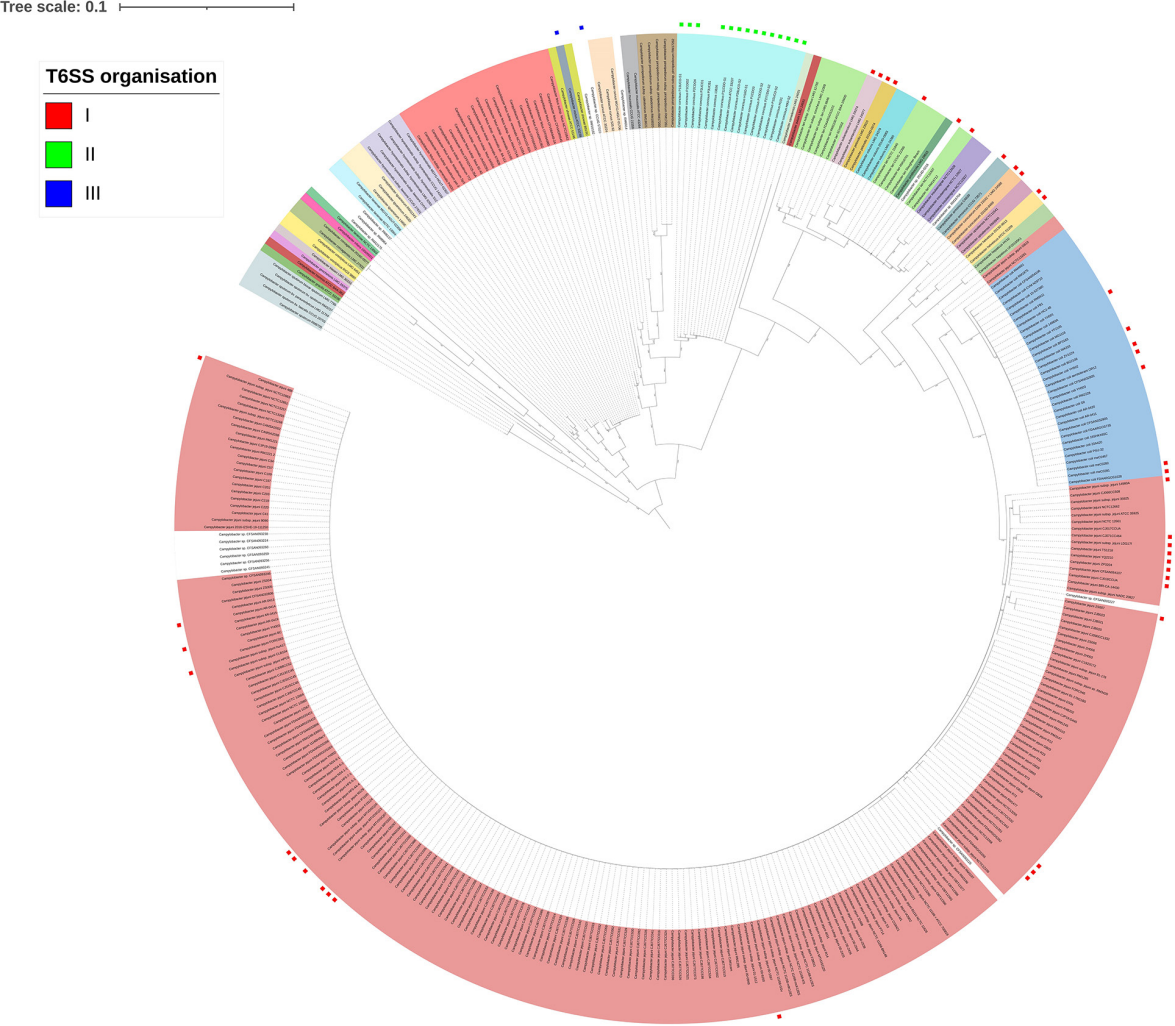
Maximum-likelihood tree based on a muscle alignment of AtpA amino acid sequences from all *Campylobacter* genomes within the dataset (FastTree2, WAG+CAT model) and visualized using iTOL [57]. The scale bar indicates the genetic distance, and the bootstrap value of each node is given. The tree is annotated with the major T6SS organizations found in distinct genomes: the red square is T6SS organization I, green is organization II and blue is organization III. Node labels are coloured to distinguish between *Campylobacter* species.

Next, we screened the 13 core T6SS loci and orphan *vgrG* locus VgrG2 from T6SS-positive *C. jejuni* 488 against the locally constructed database, identifying 62 (16.66 %) genomes to possess at least 12 of the 13 major T6SS components, and therefore classifying them as T6SS-positive (Tables S1 and S2). No T6SS-positive genomes were found to present in more than one copy of the complete T6SS cluster. *Campylobacter cuniculorum* 2010D-8469 and *C. cuniculorum* LMG 2588 were the only genomes predicted to harbour two *tagH* genes. *C. jejuni* FDAARGOS_421, NCTC13265, RM1221, RM1221 (2) and CLB104, *C. coli* AR-0411*,* and *C. concisus* P3UCB1 and P3UCO1 strains were identified to possess up to two of the screened T6SS components but were not considered to harbour a T6SS. In total, 11 species (33.33 %) in our database were identified as possessing a T6SS operon, as well as a further five strains unassigned to a known species. Again, the most prevalent species was *C. jejuni,* with 23 strains predicted to harbour a T6SS, followed by *C. concisus* (*n*=14) and *C. coli* (*n*=7) (Fig. 1c). Phylogenetic analysis of all *Campylobacter* genomes under study further highlighted the spread of the T6SSs within the genus, with distribution across the genus rather than within distinct groups (Fig. 2).

Of the T6SS-positive genomes, 46 (74.19 %) were isolated from humans as hosts, six (9.68 %) from avian species, and ten (16.13 %) from other or unknown species (defined as all species not considered human or avian in the metadata) and environmental isolation. Therefore, among the genomes under study, 16.37 % were identified as T6SS-positive within all human isolates (*n*=281), 17.65 % within all avian species isolates (*n*=34), and 17.54 % within all other species or unknown isolates (*n*=57). Among the 11 species observed to encode a T6SS, to the best of our knowledge, we putatively report the first identification of a T6SS in four species: *C. cuniculorum, Campylobacter helveticus, Campylobacter armoricus* and *C. ornithocola. C. armoricus* CA639*, C. helveticus* 2013D-9613 and ATCC 51209*,* and *C. ornithocola* LMG 29815 were predicted to harbour all 13 T6SS components (*tssA–tssM*, *hcp, vgrG* and *tagH*), whilst both *C. cuniculorum* strains, mentioned previously, harbour all 13 and an additional putative *tagH* gene. Both encoded *tagH* genes in the *C. cuniculorum* strains possessed 99.67 % nucleotide sequence identity when compared to each other.

### T6SS-positive *Campylobacter* species consist of three T6SS cluster organizations

**Fig. 3.**
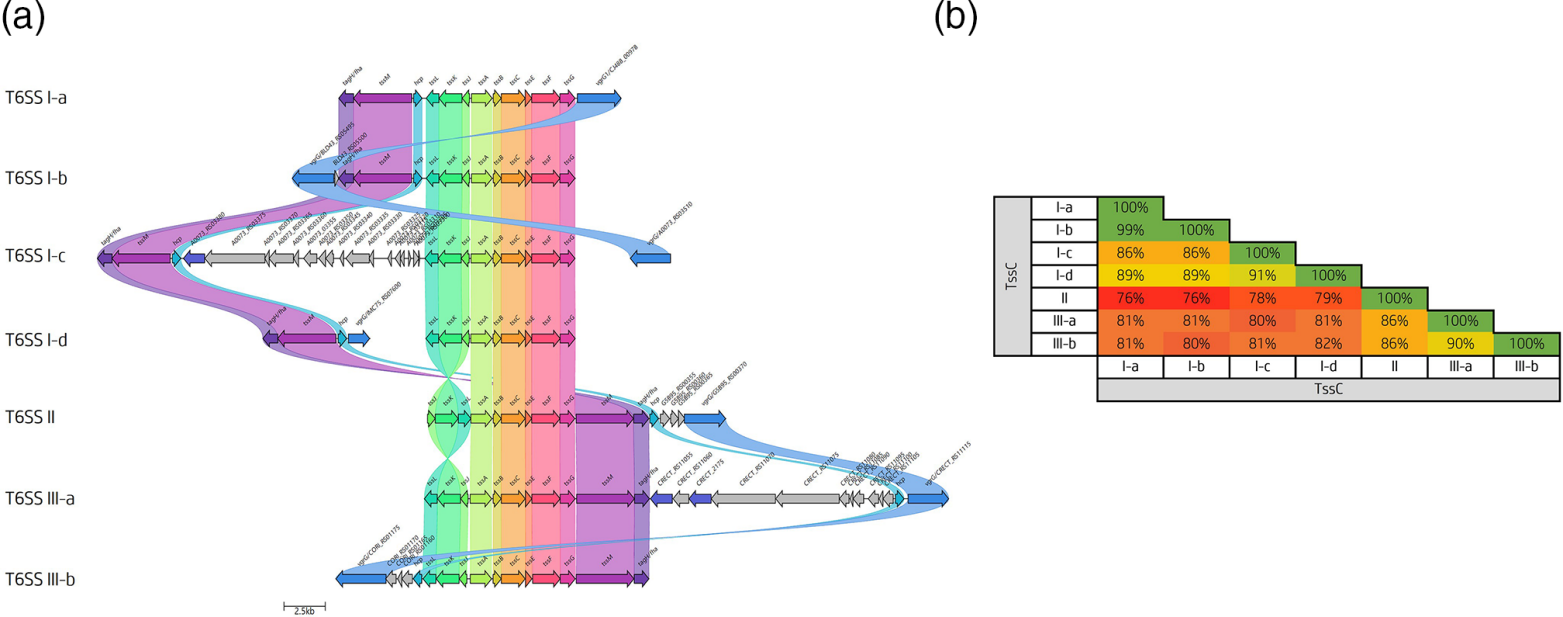
The three distinct T6SS organizations in the genus *Campylobacter *. (**a**) Comparative visualization of the genus *Campylobacter* T6SS organizations highlighting the variable nature of the operon. Genes coloured the same with connected bands are homologues. Genes found in only one genome possess a grey arrow. Arrowheads represent gene direction for transcription. Corresponding locus tags or gene names are given above the respective ORFs. The figure was generated using Clinker [50]. (**b**) Amino acid identity of the TssC homologues from the three T6SS organizations and their variants to each other. Amino acid sequence identity was calculated with blast Global Alignment–Protein (Needleman–Wunsch Global Align) [46]. The *C. jejuni* 488 strain is used as a representative genome for the T6SS I-a organization, *C. coli* ZV1224 for the I-b organization, *C. helveticus* 2013D-9613 for the I-c organization, *C. peloridis* 2016D-0074 for the I-d organization, *C. concisus* H101 for the II organization, *C. rectus* ATCC 33238 for the III-a organization and *C.* sp. CCUG 57310 for the III-b organization.

After determining the prevalence of the T6SS, we next investigated the organization of the T6SS clusters amongst the T6SS-positive genomes. During a systematic assessment of the clusters, we putatively predict three T6SS organizations (I–III) (Fig. 3a), of which two break down into further variants. T6SS organizations I-a to I-d possess a core set of nine components (*tssL* to *tssG*) which cluster together in the same orientation. Observable differences between the variants are determined by the location of the gene *vgrG* and *tagH-tssM-hcp* genetic cluster. In both I-a and I-b, the genes *tagH-tssM-hcp* are localized at the N-terminus of the main cluster adjacent to *tssL*, whilst the location of the *vgrG* varies. Alternatively, in organizations I-c and I-d, multiple genetic loci separate the main cluster from the genes *tagH-tssM-hcp,* and in the case of I-d, several *vgrG*s and effectors (see below) are harboured within this genetic region.

T6SS II possesses a unique organization that differs significantly from the T6SS I:

1. Genes *tssL-tssK-tssJ* are encoded in the reverse orientation to their homologues in the T6SS I and III organizations. As a result, all genes of the cluster are encoded in the same transcriptional direction and suggest a coregulated expression.
2. Genes *tssM-tagH-hcp* are found at the C-terminus of the main cluster adjacent to the gene *tssG.*
3. Regions of genetic variation are found between genes *hcp* and *vgrG.*

The final organizations T6SS III-a and III-b were unique to one strain in the database, respectively. The organizations of III-a and III-b both possessed genes *tssM-tagH* at the C-terminus of the main cluster, similar to T6SS II, but the orientation of *tssL-tssK-tssJ* was similar to T6SS I. Observable differences between variants III-a and III-b lie with the location of the genes *hcp* and *vgrG*. In T6SS organization III-a, the genes *hcp-vgrG* are found distant from the C-terminus of the main cluster separated by more than 10 genes, whereas in organization III-b both *hcp* and *vgrG* are encoded at the N-terminus with several genes separating *hcp* and *vgrG*.

All *C. jejuni* strains were identified to possess the T6SS organization I-a, as well as *Campylobacter* sp. CFSAN093227, CFSAN093238 and CFSAN093246, *C. cuniculorum* 2010D-8469 and LMG 2588, and all *C. coli* strains, except for strain ZV1224 which exclusively possessed organization I-b (Table S1). Organization I-c was observed in the genomes of *C. helveticus* 2013D-9613 and ATCC 51209. Genomes of strains *C. armoricus* CA639, *C. ornithocola* LMG 29815, *C. lari* RM16712, *Campylobacter* sp. 2014 D-0216, *Campylobacter peloridis* 2016D-0074 and LMG 23910, *Campylobacter subantarcticus* LMG 24374 and LMG 24377, and *C. lari* NCTC 11845 were identified to harbour the T6SS organization I-d. The number of *vgrG* genes carried by the genomes with T6SS organization I-d varied from one to five within the genetically variable region. All *C. concisus* genomes were classified to present the T6SS II organization (Fig. 2), although strain P26UCO-S2 was identified to lack the gene *tssJ* and strain P27CDO-S2 harboured a split *tssG* gene. Organizations T6SS III-a and III-b were solely observed in *C. rectus* ATCC 33238 and *Campylobacter* sp. CCUG57310, respectively (Table S1). Analysis of the core component TssC was also performed between the three T6SS organizations and their variants, confirming higher homology within the distinct T6SS organizations than to each other, suggesting the organization variants are related (Fig. 3b).

### Interspecies phylogenetic analysis of T6SS-positive *Campylobacter* species

**Fig. 4.**
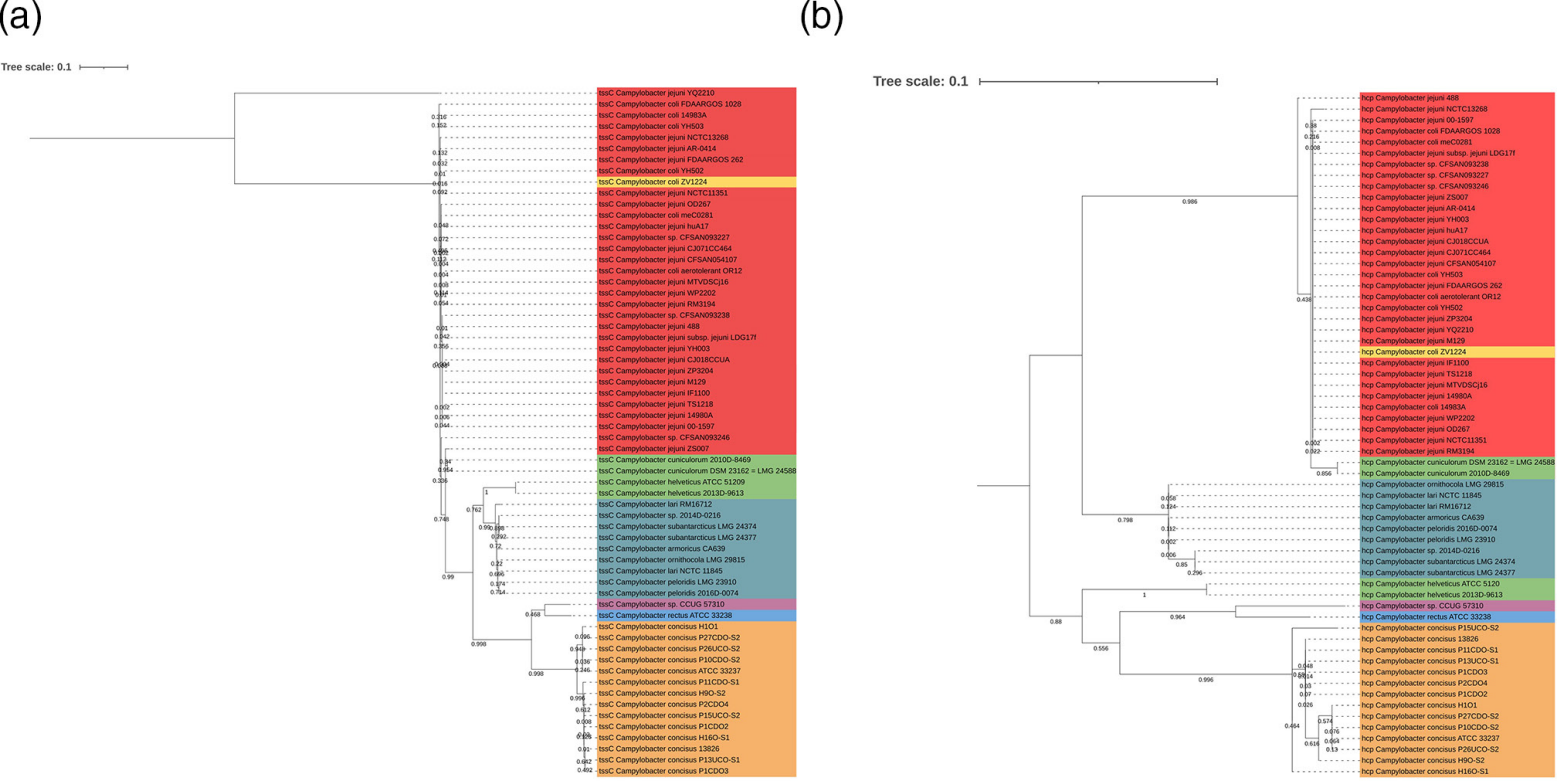
Phylogenetic trees derived from a maximum-likelihood analysis on muscle alignments of (**a**) TssC and (**b**) Hcp amino acid sequences from T6SS-positive *Campylobacter* species (bootstrap *n*=500, partial deletion) and visualized using iTOL [57]. The scale bar indicates the genetic distance, and the bootstrap value of each node is given. The node colour indicates T6SS organization: red=I-a, yellow=I-b, green=I-c, teal=I-d, orange=II, blue=III-a and burgundy=III-b.

Deletion of *tssC* in the *C. jejuni* 488 strain leads to a reduction in TssD (Hcp) secretion and thus is an important constituent of T6SS functionality [23]. Component Hcp has also been suggested as a putative effector in *C. jejuni* and is therefore likely to present species-specific traits to prevent immunity during T6SS-mediated conflict [37, 66]. We, therefore, chose to infer a phylogeny for genomes harbouring a T6SS under study (*n*=62) by producing two maximum-likelihood phylogenetic trees based on the core-sheath component TssC and secreted component Hcp as they each appear to contribute distinct roles to the activity of the *Campylobacter* T6SS. Both trees produced similar topological arrangements of T6SS-positive species and strains (Fig. 4a and b). We observed an overall high diversity within strains as several small subgroups could be identified, with clustering dependent on the T6SS cluster organization (Fig. 4a). Grouping for the Hcp phylogenetic tree also followed a similar topology but could be further clustered into two larger clades, potentially due to the absence of outliers (Fig. 4b), as observed by the sequence for TssC from *C. jejuni* YQ2210 in Fig. 4a.

### Approximately half of T6SS-positive *Campylobacter* species investigated harbour multiple VgrGs

Recently, we identified the presence of two distinct *vgrG*s harboured by the T6SS-positive strain *C. jejuni* 488 [1]. To assess the prospect of multiple *vgrG*s harboured by other T6SS-positive *Campylobacter* species, we extracted the amino acid sequences from our local protein database that matched the VgrG domain (COG3501, amino acids 45–563) of VgrG1 (CJ488_00978) from *C. jejuni* 488. In total, 113 homologous sequences were identified initially, of which 19 were subsequently removed due to criteria described in the Methods. The remaining 94 sequences were subject to further analysis. The average number of *vgrG* sequences identified amongst the T6SS-positive genomes was greater than one (*n*=1.633), with 30 (48.39 %) genomes harbouring more than one VgrG (Figure S1). Notably, five *vgrG*s were identified in *C. lari* NCTC 11845, located in the genetic region between genes *hcp* and *tssL* of T6SS organization I-d. Lengths of VgrGs also varied greatly, with *C. concisus* H9O-S2 harbouring a VgrG of 1025 aa, whilst the shortest of 430 aa was identified in multiple strains. VgrG sequences were then analysed to identify conserved and additional domains. As expected, all sequences across all strains were found to harbour the conserved domains VgrG (COG3501) and VI_Rhs_Vgr superfamily (cl37255) in the N-terminus (Table S3). Several strains were predicted to harbour the 5_superfamily (cl33691) domain previously observed in the sequence of VgrG2 from *C. jejuni* 488 [1]. Several other domains were also observed within the VgrG sequences but considered non-significant (Table S3).

### CJPI-1 is a diverse and mobile genetic island

Following on from our previous study [1], we next investigated the prevalence and diversity of the ‘CJIE3-variant’ CJPI-1 amongst the *C. jejuni* strains under study. *In silico* identification and visualization in Artemis [49] was then used to confirm the genomic location of the T6SS (i.e. genomic island or plasmid). All T6SS-positive *C. jejuni* strains (*n*=23) in our dataset harboured a T6SS either within the CJPI-1 (*n*=18) or on a plasmid (*n*=5) (Tables S1 and 4). Comparative analysis of the newly identified CJPI-1 amongst *C. jejuni* strains against CJPI-1 from *C. jejuni* strain 488 revealed considerable diversity in length and genetic composition between harbouring strains (Table 1); however, chromosomal incorporation was consistently adjacent to a tRNA^Arg^ and a query coverage of greater than 75 % was observed. In comparison, the CJIE3 from T6SS-negative *C. jejuni* RM1221 possesses a query cover of 45 % when aligned against CJPI-1 from T6SS-positive strain 488, suggesting closer genetic similarities between CJPI-1s from T6SS-positive *C. jejuni* strains than to CJIE3. Assemblies of *C. jejuni* genomes CJ018CCUA and CJ017CC64 were removed from the comparative analysis due to potential *in silico* errors. We assume minor inaccuracies in the assemblies as genes that were predicted within the putative CJPI-1 and other genomic loci were found dispersed towards the end of the assembly file. Consequently, we could not accurately predict the length, G+C content and/or position of the CJPI-1 during the comparison to strain 488.

**Table 1.** Analysis of CJPI-1s found in *C. jejuni* strains under study. Data were collected using the Artemis comparison tool (v18.1.0) [49] and blastn webtool [46]

* C. jejuni* strain ID	Length of CJPI-1 (kb)	Chromosome position (nt)	No. of genes	G+C content (%)	Genome G+C content (%)	Integrase locus	Query coverage (%)
488	~70.3	883 538–953 903	77	26.73	30.26	CJ488_0930 c	100
00-1597	~61.4	941 969–1 003 354	51	26.63	30.37	PJ17_RS04950	75
14 980A	~103.5	913 056–1 016 609	95	26.65	30.25	CJ14980A_RS04755	86
AR-0414	~74.9	827 033–901 979	68	26.95	30.36	F4V26_RS04610	96
CFSAN054107	~104.7	1 437 320–1 542 021	98	26.45	30.15	C9J79_RS07600	93
CJ018CCUA	na	na	na	na	na	na	na
CJ071CC464	na	na	na	na	na	na	na
FDAARGOS_262	~117.8	1 107 844–1 225 628	117	26.44	30.22	A6J90_05940	91
IF1100	~61.7	893 701–955 418	61	26.80	30.23	BLD34_RS04785	79
LDG17f	~92.3	975 284–1 067 564	76	26.78	30.30	E8N02_RS05125	97
M129	~79.5	936 529–1 016 065	74	26.95	30.29	CJM129_RS04930	96
MTVDSCj16	~85.7	1 010 779–1 096 499	94	26.46	30.29	MTVDSCj16_RS05475	81
NCTC11351	~115.8	940 199–1 056 036	101	26.43	30.23	AT682_RS04900	90
NCTC13268	~92.0	950 800–1 042 778	89	26.52	30.26	EL228_RS04925	92
TS1218	~88.9	950 413–1 039 331	84	26.65	30.25	BLD42_RS05120	87
YH003	~71.0	974 153–1 045 144	63	26.93	30.35	FNK00_RS05145	95
YQ2210	~92.9	930 122–1 023 004	90	26.67	30.13	BLD41_RS05290	90
ZP3204	~91.9	940 911–1 032 787	94	26.58	30.23	BLD40_RS05340	90
**Average**	88.93	na	83	26.67	30.26	na	89.88

na, Not applicable.

Twelve (5.63 %) out of 212 *C. jejuni* genomes were also identified to possess the integrative element CJIE3 (Table S1). Additionally, several non-* C. jejuni* species were putatively identified to possess synonymous T6SS-harbouring islands, as well as T6SS-encoding plasmids observed in *Campylobacter* sp. CFSAN093246 and *C. coli* strains 14 983A, FDAARGOS_1028, meC0281, YH502 and YH503.

### T6SS-positive *Campylobacter* species encode multiple putative effector subsets neighbouring VgrGs

Recently identified within CJPI-1 of *C. jejuni* 488, four putative effectors and two immunity genes were predicted to be encoded within a genetically variable region between the genes *vgrG1* and *vgrG2,* representing the first potential secreted substrates to be utilized by the T6SS in *Campylobacter* [1]. We hypothesized that other effectors and secreted proteins could be found neighbouring *vgrG*s in the previously identified T6SS-harbouring *Campylobacter* species. To achieve this, we systematically extracted the protein sequences of the five upstream (+5) and downstream (−5) genes adjacent to identified *vgrG* homologues across all T6SS-positive species under study. Extracted sequences were then subject to functional characterization through domain, signal peptide and transmembrane helix analyses (Table S5).

**Fig. 5.**
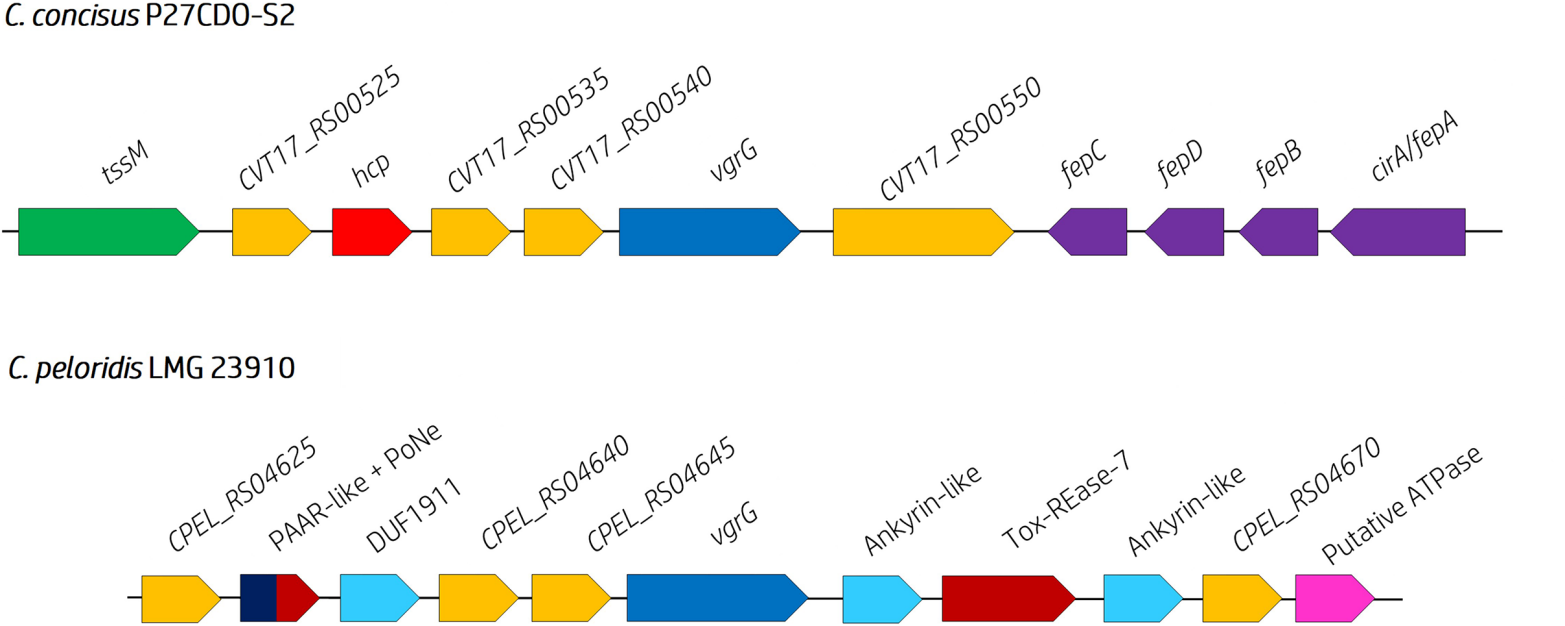
Genomic organization and categorization of select genes +/−5 upstream and downstream of *vgrG*s in the T6SS-positive *Campylobacter* genomes under study. VgrGs are coloured blue, TssM are green, Hcp are red, hypothetical genes are orange, immunity genes are light blue, toxin domains/putative effectors are red, PAAR-like domains are dark blue, iron ABC-binding cassette system components are purple and ATPase are pink. Corresponding gene names and predicted functions are given above the respective arrow. Species and strain IDs are also given. Genes are not to scale. The orientation of an ORF is dictated by the direction of the arrow (forward=right, reverse=left).

We predicted two effector–immunity pairs amongst the T6SS-harbouring population. The first pair consisted of proteins harbouring domains belonging to the Ankyrin (PF12796/cl33707) and Tox-REase-7 (cl21441) superfamilies (e.g. CJM129_RS05125 and CJM129_RS05130 in *C. jejuni* M129) and was commonly found downstream of the cognate *vgrG*. The second pair encoded domains belonging to the PoNe (cl41756) and DUF1911 (cl07503) superfamilies (e.g. A6J90_RS09495 and A6J90_RS06400 in *C. jejuni* FDAARGOS_262) and was identified upstream of the cognate *vgrG* (Fig. 5). Both effector domains are characterized to act as nucleases [67, 68]. Genes coding for Tox-REase-7-containing proteins were observed in the genomes of 14 *C. jejuni* strains, six *C. coli* strains, *C.* sp. 2014D-0216, *C. peloridis* 2016D-0074 and LMG 23910, *C.* sp. CFSAN093227, *C. subantarcticus* LMG 24374 and LMG 24377, and *C. lari* NCTC11845. PoNe domain-containing proteins were identified in the genomes of four *C. jejuni* strains, *C.* sp. 2014D-0216, *C. peloridis* 2016D-0074 and LMG 23910, and *C. subantarcticus* LMG 24374 and LMG 24377 (Table S5). Other putative effectors were also frequently observed near the *vgrG*s encoding domains belonging to the Tox-AHH (PF14412), lysozyme (cl13324), endolysin (cl00222), peptidoglycan hydrolase (cd12797) and lipase (cl21494) families (Table S5). Tox-AHH domain-containing proteins encoded by +5 or −5 genes were observed in the genomes of 10 *C. jejuni* strains, five *C. coli* strains, *C.* sp. CFSAN093227 and *C.* sp. CFSAN093238, and *C. lari* NCTC11845 and RM16712. Lysozyme/endolysin domain-containing proteins were predicted in the genomes of four *C. concisus* strains and *C.* sp. CCUG 57310. Interestingly, *C.* sp. CCUG 57310 was also predicted to encode a lysozyme immunity (cl34764) domain-containing protein, CORI_RS01180, adjacently upstream of its lysozyme (cl00222) domain-containing protein, CORI_RS01185. Additionally, five putative ATPase- (cl34796) encoding proteins were also predicted near *vgrG*s in the genomes of *C. peloridis* 2016D-0074 and LMG 23910 and *C. lari* NCTC11845, as well as putative ATP-binding cassette (ABC) Fe^3+^-siderophore transport system (cl00262, COG1120, PF01032 and cl36974/COG4771) domain-containing proteins in *C. concisus* P27CDO-S2 (Fig. 5), and FepB protein encoding genes in four other *C. concisus* strains (Table S5).

Of note, the most frequently observed proteins that encoded identifiable domains (*n*=89) either −1 or +1 genes neighbouring *vgrG*s were predicted as Ankyrin domain-containing (37.08 %, *n*=33), TssG (34.83 %, *n*=31) or Hcp (11.23 %, *n*=10) proteins. Similarly, predicted proteins encoded by either +2 or −2 genes with identifiable domains (*n*=85) were predicted as TssF (36.74 %, *n*=31), Tox-REase-7 (31.76 %, *n*=27), TssM (10.59 %, *n*=9) or Lysozyme/Endolysin (5.88 %, *n*=5) proteins (Table S5).

### A PAAR-like domain is widespread amongst T6SS-harbouring *Campylobacter* species

A bioinformatic screening for the PAAR-like domain identified in the protein CJ488_0990 (amino acids 25–150) from *C. jejuni* 488 [1] was also conducted against the local *Campylobacter* database to assess its prevalence. As a result, 240 homologous protein sequences were predicted to harbour the domain across the *Campylobacter* genomes, including both T6SS-harbouring and T6SS-absent genomes (Table S6). This was further confirmed by aligning the homologous sequences and visualizing the presence of the conserved cysteine and histidine residues (Fig. S2). The PAAR-like domain-containing sequences were then analysed for additional domains using NCBI-CDD and Pfam [51, 63]. Several proteins were subsequently found to harbour additional toxic domains. The toxin domain TNT (PF14021, *n*=14) and PoNe (cl41756, *n*=19) superfamily were consistently found in the C-terminus of select PAAR-like domain-containing proteins (Fig. 5). Further, several PAAR-like domain-containing proteins harboured a lipase (cd00519, *n*=19) superfamily in their N-terminus.

PAAR-like domain-containing proteins identified in the previous stage of analysis were also cross-referenced with the genes extracted from +5 upstream to −5 downstream of the *vgrG*s. Sixteen proteins encoded by genes neighbouring *vgrG*s were predicted to contain the PAAR-like domain, of which nine (56.25 %) also possessed C-terminal PoNe toxin domains. The PAAR-like domain-containing proteins encoded by genes +5 to −5 of the identified *vgrGs* were found in the genomes of *C. jejuni* 14 980A, FDAARGOS_262, NCTC11351 and NCTC13268, *C. peloridis* 2016D-0074 and LMG 23910, *C. lari* NCTC11845 and RM16712, and *C. subantarcticus* LMG 24377 (Table S5).

## Discussion

The T6SS is a multifunctional weapon with a broad range of targets due to its array of deployable toxic effectors [12, 69]. The function of the *Campylobacter* T6SS has been explored in *C. jejuni,* contributing to different physiological mechanisms to promote pathogenesis [25, 41]. In this study, we identified within a subset of publicly available *Campylobacter* genomes that 33 % of *Campylobacter* species under study putatively possess a T6SS. We note that *Campylobacter ureolyticus* DSMZ 20703 was previously reported to encode homologues of T6SS components [40], but it was not present in the assembly data downloaded from NCBI and thus not investigated. We also note that several recently identified novel species of the genus *Campylobacter* were also absent from the NCBI RefSeq release 205 database, including *Campylobacter aviculae, Campylobacter estrildidarum, Campylobacter taeniopygiae* [70]*, Campylobacter portucalensis* [71]*, Campylobacter novaezeelandiae* [72]*, Campylobacter massiliensis* [73] and *Campylobacter vulpis* [74], of which two were discovered after the database was downloaded. As such, our database does not encompass all *Campylobacter* species to date, and therefore we stress that the results of our study are exclusive to species only included in the local database.

It is also important to state that although many T6SS-harbouring species included in this analysis possess only one sequenced isolate, we cannot consider the genomes to be representative of the species. Variation in intraspecific T6SS prevalence within *Campylobacter* species is evident [1, 75, 76]; therefore, we recommend not to consider these isolates as ‘representative’ of the species but included them only to incite further exploration into the presence of the T6SS amongst *Campylobacter* species. Moreover, the reverse can also be stated for the absence of a T6SS in select species. Species and strains other than those included in our ‘complete’ genome dataset may possess a T6SS, such as the recently identified *Campylobacter* species mentioned above, warranting further investigation. Unsurprisingly, our study demonstrates the presence of the T6SS within *Campylobacter* is not solely restricted to one or two species, similarly to other T6SS-harbouring genera [15, 77]. Its widespread presence has probably been achieved by events of genetic acquisition [78, 79]. Ultimately, this would independently lead to the same beneficial traits for distinct survival motives, such as competition or niche survival [80].

Since 2012, seven *Campylobacter* species. have been identified to encode a complete T6SS [37–40, 43]. Here, we add a further four with the prediction of a T6SS in *C. cuniculorum, C. helveticus, C. armoricus* and *C. ornithocola.* We hope that reporting the presence of a T6SS in these species advances the knowledge about their putative virulence, in addition to developing strategies to control their distribution and pathogenicity. We also anticipate this novel finding to be the ‘tip of the iceberg’ for *Campylobacter* as more ‘emerging’ strains are sequenced and investigation into T6SS prevalence is conducted. Interestingly, both *C. cuniculorum* strains predicted to harbour a complete T6SS cluster were observed to encode an additional *tagH* gene. The function of this gene has not been independently characterized in *Campylobacter* species [22], but its possession of a forkhead-associated (FHA) domain (COG3456) suggests it could potentially play a regulatory role, as seen in *Pseudomonas aeruginosa* [81].

Prevalence of the T6SS in *C. jejuni* isolates from humans has been observed to vary between low- and middle-income and high-income countries [76]. In this study, T6SS-harbouring isolates were observed predominantly with humans as the source of isolation. However, it is important to note that this observed association is probably due to the partiality to sample and upload of genomes of clinically relevant isolates, such as those from humans, rather than environmental isolates; therefore, over-representation of their prevalence in our dataset is likely. This is also in addition to potential sequencing bias of isolates from other non-human species prohibiting the inference of T6SS prevalence in different hosts. Further research using isolates from singular hosts is needed to explore the associations between the *Campylobacter* T6SS and humans, as well as disease severity.

Numerous T6SS-harbouring genera possess several T6SSs within their genomes clustered into distinct organizations [82, 83]. Here, we predict three distinct genetic organizations encoding the components for a singular T6SS cluster within *Campylobacter *. Discrepancies between the T6SS variant organizations of I and III imply genetic reshuffling, potentially arbitrated by horizontal gene transfer, has allowed translocation of the *vgrG* gene. This may have occurred through its possession of a ‘rearrangement hotspot’ element, previously observed in *C. jejuni* [22]. Phylogenetic analysis of all *Campylobacter* genomes under study and T6SS-positive isolates also highlighted the conservation of T6SS cluster organizations within distinct species. T6SS organization II and variants of organization III were exclusively found in one species, respectively, whereas T6SS organization I was identified in 10 species (Figs. 2 and 4). To deduce why particular organizations are favoured by specific *Campylobacter* species is beyond the scope of this study, but factors such as the source of isolation (i.e. host), genomic location (e.g. chromosome, plasmid), effector utilization and conservation of energy may contribute to their determination. T6SS organization II potentially demonstrates this prospective benefit of energy conservation by encoding its T6SS genes in the same transcriptional direction, thus relying on a singular promoter to co-regulate the expression of the entire T6SS cluster as one operon. Furthermore, as observed in this study and our previous work, T6SS cluster-containing mobile genetic elements could also lead to the dissemination of organizations among species with homologous recombination potentially facilitating chromosomal integration [1].

Encoding multiple T6SS clusters also leads to the presence of several structural component homologues across genomes. T6SS-harbouring species can encode multiple *vgrG*s and *hcp*s distributed between main and orphan clusters, exhibiting diverse functions [12, 19, 67, 84]. Two distinct *vgrG* genes were putatively identified by our group in the CJPI-1 of the *C. jejuni* 488 strain, with homologues to both in *C. coli* genomes [1]. Although all T6SS-harbouring species were observed to possess one T6SS cluster, our prevalence study identified a significant proportion of the T6SS-harbouring species encoding two or more *vgrG*s, as well as a conserved domain across all VgrG protein sequences. All additional *vgrG*s were found distant from the main cluster. We speculate that harbouring diverse VgrGs within the genus *Campylobacter* provides unique advantages to encoding species and strains, particularly related to the diverse environments they inhabit. Typically, interchange of the spike complex VgrG trimer leads to the specific interaction of effectors via the protein’s C-termini, widening the range of cellular targets [12].

Pathogenicity islands are a unique subset of mobile genetic elements, conferring specific virulence phenotypes to harbouring bacteria governed by the genes carried [85]. In this study, we sought to verify the prevalence of CJPI-1 amongst our identified T6SS-positive *C. jejuni* population, in conjunction with exploring its prospective variation and make-up. Through comparative approaches, we found that 18 T6SS-harbouring *C. jejuni* strains encoded their T6SS within the CJPI-1, with the remainder harbouring theirs on discrete plasmids. We note considerable variation in the length and genetic composition of CJPI-1 between the strains under study, suggesting multiple events of genetic exchange have resulted in the acquisition or loss of specific genes. A select few were observed to possess additional toxin–antitoxin (TA) modules (data not shown) not detected in our previous study, whilst others harboured supplemental effector–immunity pairs in the downstream genetically variable region [1]. TA modules are prominent modulators of mobile genetic element persistence [86]; accordingly, encoding several TA modules within CJPI-1 suggests an important role. Either CJPI-1 is a significant constituent for the survival of harbouring strains and the cognate TA modules are responsible for its stabilization and fitness, or the TA modules are accumulated for other functions, namely utilization by the T6SS. Interestingly, we also noted several other *Campylobacter* species to harbour mobile genetic elements and plasmids that code for T6SSs, meriting further investigation.

Genes neighbouring *vgrG*s are frequently identified as secreted substrates for the cognate spike proteins, presenting a strategic investigative route to uncover associated toxic effectors [28, 67]. After systematically extracting the upstream and downstream protein sequences of genes adjacent to the *Campylobacter vgrG*s identified, we observed several putative effector and immunity proteins. Predicted effectors encoded nuclease domains Tox-REase-7, Tox-AHH and PoNe, as well as lysozyme/endolysin and lipase domains. These nuclease domains have been previously identified in several polymorphic protein delivery systems, including the T6SS [67, 68]. Immunity proteins were then confidently predicted adjacent to two of the effector-encoding genes, Tox-REase-7 and PoNe, with the PoNe-DUF1911 pair expanding upon the current repertoire of predicted effectors present within *C. jejuni* strains [1].

In addition to the predicted effectors, we also identified a putative ABC Fe^3+^-siderophore import system immediately downstream of a cognate *vgrG* (CVT17_RS00555 – CVT17_RS00570) in *C. concisus* strain P27CDO-S2 (Fig. 5). Investigation into the domains harboured by the proteins predicted the existence of a putative FepBCD ferrienterobactin ABC transporter system, commonly found in *Escherichia coli *, and a CirA (COG1629)/FepA (COG4771) ligand-gated outer membrane receptor protein [87, 88]. To date, several secreted metal scavenging proteins are described for the T6SS, including TseF in *P. aeruginosa* which facilities iron acquisition [89, 90]. Iron is an abundant and essential element involved in both metabolic and cellular processes. As such, insoluble ferric iron (Fe^3+^) acquisition is a vital component of iron homeostasis in bacteria [91]. During periods of iron deficiency or competition for resources, Fe^3+^ acquisition strategies commonly involve the secretion of ferric-chelating siderophores and internal translocation via receptor uptake systems [92, 93]. Several ferric uptake systems are reported in *Campylobacter *, such as the CfrA/CeuBCDE system in *C. jejuni* [94]. To the best of our knowledge, this is the first report of a putative FepABCD uptake system in the genus *Campylobacter *. Periplasmic binding protein FepB was also found downstream of *vgrG*s in four other *C. concisus* strains under study, but the permease, ATPase and outer membrane receptor were not. It remains unknown how *C. concisus* utilizes this ferric uptake system, as well as which ferric-siderophore is responsible for environmental Fe^3+^ acquisition. One hypothesis is that the *C. concisus* FepABCD system is scavenging siderophores produced by other competing bacteria. On the other hand, it is also possible that a distant chromosomal gene to the *vgrG* is encoding the metal scavenging siderophore, potentially secreted by the cognate secretion system; however, it is important to note that the FepABCD system is encoded in the opposing transcriptional orientation to the *vgrG*.

PAAR domains are encoded in several bacterial pathogens, frequently harboured in the N-terminus of proteins with C-terminal toxin domains [20, 95]. A PAAR-like domain-containing protein encoded in the CJPI-1 of T6SS-positive *C. jejuni* 488 [1] was screened against our local *Campylobacter* database to investigate its distribution and genetic context. We identified the presence of the PAAR-like domain across several species and strains within the genus *Campylobacter *, with a subdivision encoding toxic domains in their C-termini and others harbouring lipase domains at their N-termini. The existence of this domain across T6SS-positive species indicates a shared function of PAAR-like-containing proteins, further supported by the presence of conserved cysteine and histidine residues. A PolC (cl35100) superfamily domain, associated with the gram-positive bacterial DNA polymerase III gene *polC* [96], was also identified in 53 sequences presenting the PAAR-like domain; however, cross-checking in Pfam, due to the uncharacteristic function of the domain in conjunction with the role of PAAR and weak E-value, did not identify the domain using the predictive program. For this reason, we infer that the presence of this domain amongst PAAR-like domain-containing proteins is unlikely but warrants further investigation. We predict that the *Campylobacter* PAAR-like domain-containing protein is fulfilling both the role of sharpening the spike complex and simultaneously facilitating the transport of toxin domains to the secretion apparatus [20].

## Conclusion

In summary, this study provides the most in-depth analysis of T6SS prevalence and diversity within the genus *Campylobacter* to date. We demonstrate genetic similarities and differences between the T6SS of *Campylobacter* species, as well as variation in the number of *vgrG*s, PAAR-like proteins and putative effectors harboured by T6SS-containing genomes. We also report the putative first identification of a T6SS in four *Campylobacter* species, with other T6SS-harbouring species belonging to the growing group of ‘emerging *Campylobacter* pathogens’. Understanding the arsenal of effectors this secretion system utilizes and the mechanisms by which the apparatus can be genetically transferred will provide a foundation for future experiments characterizing the *Campylobacter* T6SS.

## Supplementary Data

Supplementary material 1

Supplementary material 2

Supplementary material 3

Supplementary material 4

Supplementary material 5

Supplementary material 6

Supplementary material 7
