# Supplementary material for: In silico investigation of the genus Campylobacter type VI secretion system reveals genetic diversity in organization and putative effectors
Source: Microb Genom. 2022 Oct 31;8(10):mgen000898. doi: 10.1099/mgen.0.000898 (PMC9676060; doi:10.1099/mgen.0.000898)
Supplement: Supplementary material 1 [file mgen-8-898-s001.pdf]

Supplementary Figure S1

Supplementary Figure S2

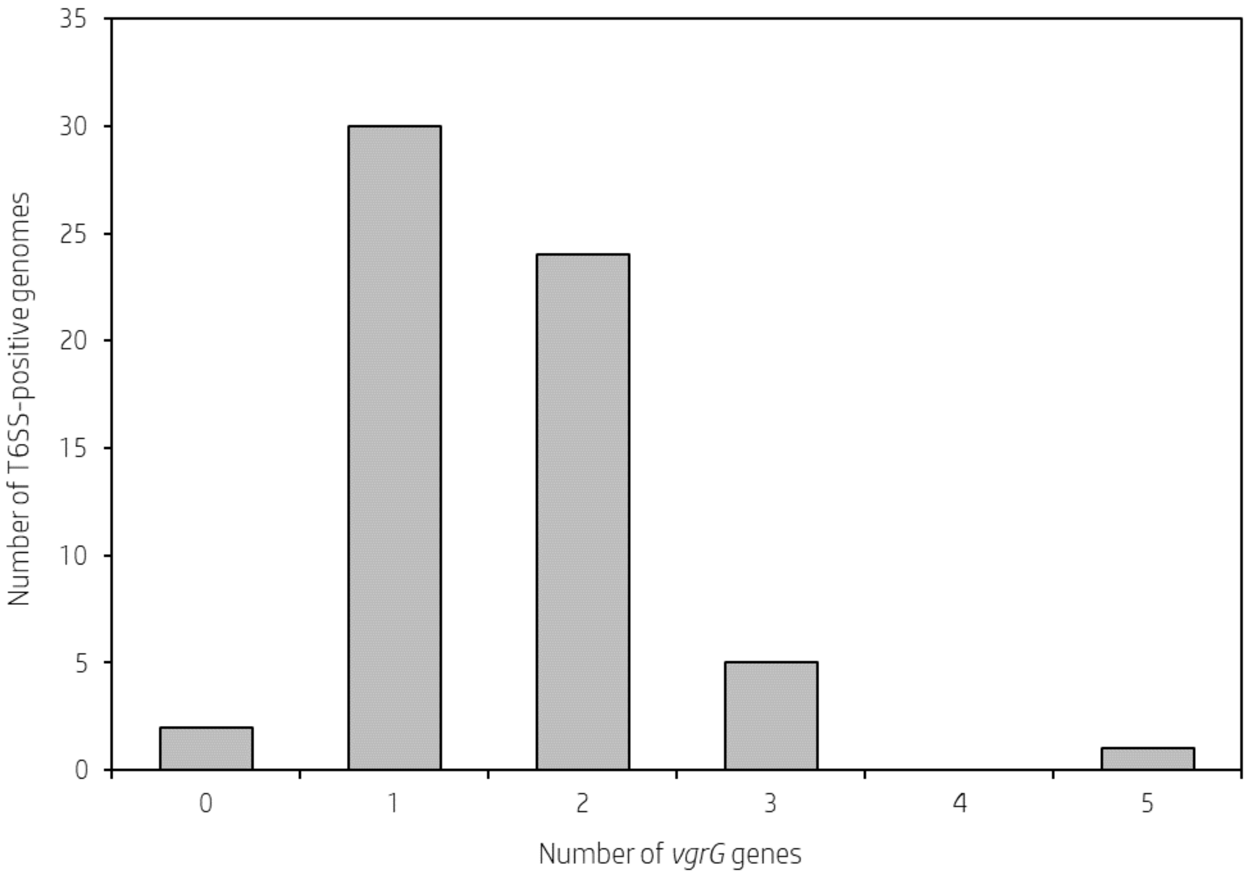

Supplementary Figure S1. The distribution of *vgrG* genes among T6SS-positive *Campylobacter* genomes. Bar graph showing the number of T6SS-positive *Campylobacter* genomes to encode between 0 – 5 *vgrG* genes.

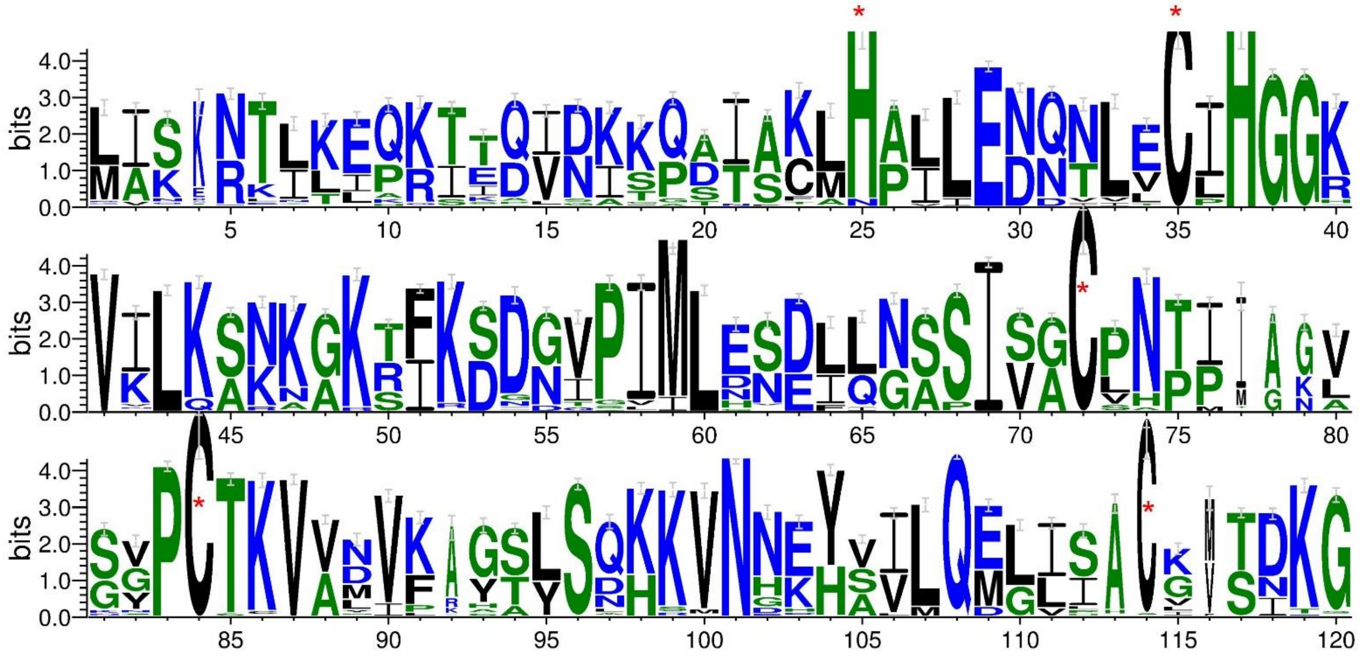

Supplementary Figure S2. Conserved residues found in PAAR-like homologs identified by ClustalOmega (Madeira *et al.*, 2019) multiple sequence alignment, showing cysteine and histidine residues for putative PAAR-like activity. Conserved cysteine and histidine residues are labelled with a red star (*). Visualised using WebLogo3 (Crooks *et al.*, 2004).
